# Microscale and Macroscale Deformation Behavior of Electrospun Polymeric Nanofiber Membranes Using In Situ SEM during Mechanical Testing

**DOI:** 10.3390/polym15071630

**Published:** 2023-03-24

**Authors:** Olivier Verschatse, Eva Loccufier, Bianca Swanckaert, Karen De Clerck, Lode Daelemans

**Affiliations:** Department of Materials, Textiles and Chemical Engineering (MaTCh), Faculty of Engineering and Architecture, Ghent University, Technologiepark 70A, 9052 Ghent, Belgium

**Keywords:** mechanical behavior, polymeric nanofiber membranes, electrospinning, in situ SEM analysis

## Abstract

Electrospun nanofiber membranes show high potential in various application fields (e.g., filtration, catalysis, and sensing). Nevertheless, knowledge of the mechanical behavior, and more specifically, the deformation of nanofiber membranes is still limited today which can complicate the appliance of nanofiber membranes in applications where they are mechanically loaded. In this paper, we, therefore, analyzed the mechanical behavior of polymeric nanofiber membranes with different fiber orientations (random and aligned) extensively. Polyamide 6 was used as a representative reference polymer for proof-of-concept. Mechanical tests show that all membranes have a coherent deformation behavior at the macroscale up to the point of fracture. Large variations in stiffness, ultimate strength, and ultimate strain were observed between membranes with different fiber orientations (Random: E-mod: 370 ± 34 MP; UTS: 38.5 ± 6.0 MPa; ε_max_: 30.0 ± 2.8%; Parallel aligned: E-mod: 753 ± 11 MPa; UTS: 55.4 ± 0.8 MPa; ε_max_: 12.0 ± 0.1%; Perpendicular aligned: E-mod: 24.1 ± 3.7 MPa; UTS:/; ε_max_: >40%). This shows the versatility and tunability of the mechanical behavior of these nanofiber membranes. At the microscale, the fibrous structure results in deformation mechanisms that resist failure formation and progression when the membrane is mechanically loaded. This results in a high fracture resistance, even for pre-damaged membranes. Realignment of the fibers along the loading direction causes crack tip blunting, locally reinforcing the membrane.

## 1. Introduction

Electrospun nanofiber membranes are a relatively novel class of materials consisting of a non-woven assembly of very thin fibers, usually with a sub-micron diameter. Both the low fiber diameter and the non-woven architecture of the membranes give them interesting characteristics, such as a high (internal) surface area and high porosity while remaining flexible [1,2,3,4]. Therefore, these materials have great potential for high-demanding applications, such as gas and water filtration, tissue scaffold engineering, wound healing, catalysis, composites, and food packaging [3,5,6,7,8,9,10,11]. However, the successful application of these materials often also require good mechanical performance and endurance [12].

Although electrospun membranes can be regarded as non-woven, i.e., the (nano)fibers forming the membrane are randomly dispersed in the plane of the membrane, they resemble a continuous film material at the macroscale since the fibers cannot be identified without adequate microscopy. Many researchers have performed introductory mechanical testing such as classical tensile tests or DMA tests on the membranes to have a macroscopic view of the material’s performance [13,14,15,16,17,18,19,20,21]. This approach treats the material as a continuous film, and thus gives a good comprehension of the overall performance of the membranes. However, it lacks insight into the deformation mechanisms happening at the fiber level. For example, while ductility is observed for membranes made from ductile polymers (e.g., polyamides), the question remains whether this ductility of the membrane can be completely attributed to the polymer choice. It is expected that the fibrous architecture of this non-woven plays a significant role in the material’s response, since the fibers can realign, slip, and stretch at the microscale, having an effect at the macroscale beyond the properties of the raw material as such [22,23]. Maccaferri et al. showed that the amount of contact points between the nanofiber in the membrane has an influence on the overall mechanical behavior of the membrane [24]. Andersson et al. observed the necking of individual nanofibers during the loading of the nanofiber membrane [25]. These examples show that a good understanding of what happens at the microscale level is needed to completely capture the phenomena observed at the macroscale level. Yet, a complete understanding of the deformation behavior of electrospun polymeric nanofiber membranes, linking this dual micro- and macroscale nature, is thus lacking today, especially the influence of fiber orientation and pre-imposed damage (line ruptures, holes, etc.) on the membrane’s mechanical response. In this respect, the use of in situ electron microscopy can reveal what happens at the microscale. However, only a few studies used this technique to date [25,26,27].

**Figure 1:**
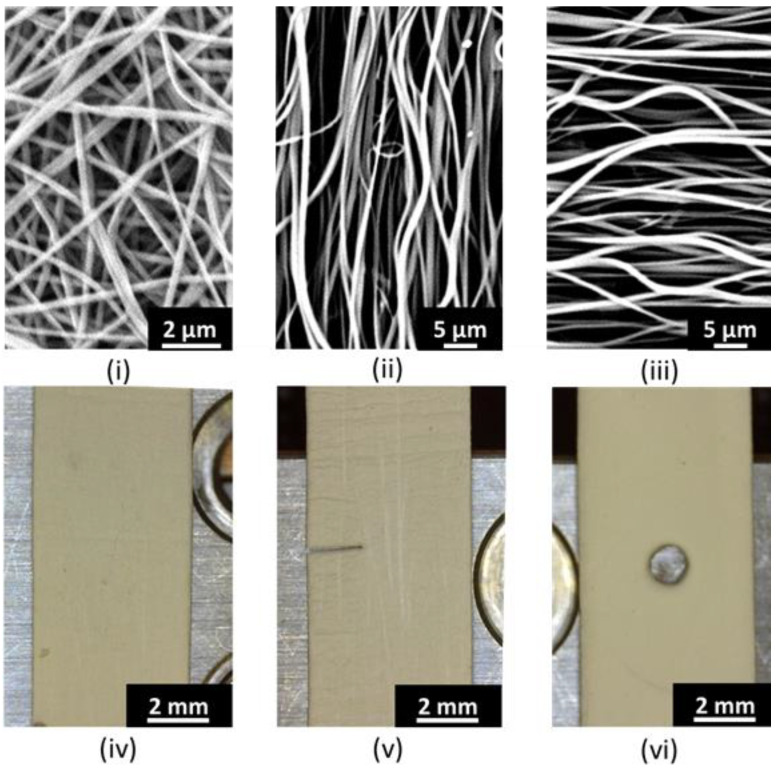
PA6 membranes studied within this work. Three different nanofiber orientations compared to the tensile direction are considered: (**i**) random, (**ii**) parallel, and (**iii**) perpendicular. Three specimen types were considered for every fiber orientation: (**iv**) pristine, (**v**) line rupture, and (**vi**) hole perforation.

The present work focuses on an in-depth study of the deformation and failure behavior of electrospun polyamide-6 (PA6) using in situ scanning electron microscopy (SEM) during mechanical loading to understand the dual micro- and macroscale nature of this material class. In situ SEM during mechanical loading allows linking the macroscopic imposed loading to the deformation mechanisms at the microscale, i.e., the scale of the nanofibers. This analysis is performed on both pristine as well as notched/pre-damaged specimens each time for random and oriented (parallel and perpendicular to the loading direction) nanofibers (Figure 1) to analyze the effect of defects on the material’s performance and endurance. In situ SEM observation of the pre-damaged sample can give us more information on the internal structure and fiber rearrangement of the nanofiber membranes. PA6 nanofiber membranes are selected, since these are very common in electrospinning research and applications [28,29,30,31,32]. PA6 is relatively simple to electrospun and has been used in many applications such as air filtration [29] and for batteries [30]. Furthermore, the PA6 polymer has a representative mechanical behavior similar to many other polymers. Therefore, PA6 nanofiber membranes are selected in this work as a model system for polymeric nanofiber applications. The combination of both pristine and pre-damaged specimens, together with a follow-up of the deformation behavior with in situ electron microscopy results in important insights into the performance of nanofiber structures. In addition, it is proven to be a viable testing framework for this material class.

## 2. Materials and Methods

### 2.1. Electrospinning

Polyamide 6 (Sigma Aldrich (St. Louis, MO, USA), Mw 51,000 g mol^−1^), formic acid (Sigma Aldrich (Darmstadt, Germany), 98%), and acetic acid (Sigma Aldrich (Espoo, Finland), 98%) were used as received in a 1/1 volume-based formic acid (FA)/acetic acid (AA) solvent system. An amount of 18 wt% of polyamide 6 (PA6) was added at room temperature using a magnetic stirrer to obtain a clear and stable electrospinning solution, as reported in detail in previous studies [33,34,35]. For the production of homogeneous membranes of a certain thickness, a single nozzle (ID 0.4 mm, OD 0.8 mm) that moved linearly along/parallel to a collector was used. For the random oriented membranes, a conveyer belt setup was used at a rolling speed of 0.1 mm/min while the nozzle moved over a width of 15 cm. The polymer solution was fed to the needle tip at a rate of 3.5 mL h^−1^, and a total of 3 nozzles were used to increase the production rate. A high voltage power supply (Glassman High Voltage Series) was used at 30 kV to apply a voltage difference between the needle tip and the collector, which were separated by a distance of 8 cm. For the aligned membranes, a rotating drum collector (OD 12 cm, length 50 cm) was used at high speed (60 Hz resulting in a tangential speed of 22.6 m/s) to ensure the orientation of the nanofibers. The polymer solution was fed to the nozzle at a rate of 2.5 mL h^−1^. A high voltage power supply (Glassman High Voltage Series) was used at 25 kV to apply a voltage difference between the nozzle and the collector, which were separated by a distance of 10 cm. A difference in diameter was noted between the random and aligned membranes (Figure 1, nanofiber diameter random: 250 ± 60 nm, aligned: 420 ± 95 nm (coagulated nanofibers were left out where possible)), which is likely due to the different settings used in each production system.

### 2.2. Sample Preparation

The produced membranes have a nominal size of 200 mm × 300 mm, of which test specimens of 5 mm × 30 mm were cut. For each specimen, the areal weight was determined by measuring the mass on an analytical balance (precision of 2 µg). The nominal areal weight of the produced membranes was 38.5 ± 2 g/m^2^ (thickness of 0.18 ± 0.02 mm) for the random oriented membranes and 20.5 ± 1 g/m^2^ (thickness of 0.06 ± 0.003 mm) for the aligned membranes. The membrane thickness was measured with a micrometer with 1µm precision. Afterwards, paper tabs were attached to the specimen ends to minimize clamping damage in the tensile experiments, resulting in an effective gauge length of 20 mm for each specimen.

A total of 3 different specimen configurations were used for both random and aligned (parallel and perpendicular to loading direction) nanofiber membranes (Figure 1i–iii): a rectangular specimen, a rectangular specimen with a slit of approximately 1/3th of the total sample width at one of its sides, and a rectangular specimen with a punched hole of 1 mm diameter in the middle of the sample (Figure 1iv–vi). For the perpendicular aligned specimens with a central hole, the gauge length between the bonded tabs was reduced to 10 mm to increase the total strain level to which the specimens could be stretched.

### 2.3. Mechanical Testing

All samples were tested on a small-scale universal testing fixture (Phenom XL tensile stage) equipped with a load cell of 150 N designed to fit in a Phenom XL Scanning Electron Microscope (SEM). A constant strain rate of 2.5% min^−1^ was applied, while force and displacement were recorded. A strain rate of 2.5% min^−1^ was selected to enable semi-continuous (step-and-shoot) or even continuous in situ SEM mechanical testing while taking snapshots. To ensure relatability between tests, a strain rate of 2.5% min^−1^ was aimed for across all tests. For each specimen type in Figure 1, three tensile tests were performed.

The tests were monitored in time using a digital microscope (Dino lite AM4515ZTL). For each specimen type, a tensile test was performed while visualizing its microscale deformation behavior using SEM. These specimens were sputter-coated with a gold layer of 10 nm. The SEM images were taken via a step-and-shoot method at several preselected values of strain, to record high-quality SEM images (typical image acquisition time of 7 s).

To obtain a global overview of the strains present in the sample, mechanical tests were also performed with membrane samples containing a DIC speckle pattern on the surface (for each specimen one test; data not used for mechanical analysis). These tests were performed on samples with a gauge length of 10 mm and at a strain rate of 2% min^−1^.

### 2.4. Data Analysis

As mentioned in the introduction, nanofiber membranes are dense and coherent structures. Yet, high porosity is present in these structures (>90%) [36,37,38,39,40]. Therefore, the use of membrane width and thickness will typically result in a very low estimate of the stress present in the membrane compared to traditional solid membranes. Therefore, we compensate for the porosity of the membrane by a correction factor ($f_{correction}$):

(1) fcorrection=VnanofiberVmembrane=mnanofiberρnanofiberlmembrane×bmembrane×tmembrane 

with $\rho_{nanofiber}$ the density of the nanofiber polymer, *m_nanofiber_* the mass of the nanofiber membrane, and l, b, and t the length, width, and thickness of the membrane, respectively. This results in a corrected cross-section and a more representative value of the stress (Equation (2)):

(2) $$\sigma =\frac{F}{\left(A_{Cross-section\;Membrane}\times f_{Correction}\right)}\;$$

When the cross-sectional area *A_Cross-section membrane_* is replaced by *t_membrane_* × *w_membrane_* the formula results in Equation (3):

(3) $$\sigma =\rho_{nanofiber}\frac{F}{m_{membrane}}L_{membrane}\;$$

Equation (3) is also the proposed formula by Maccaferri et al. who did an extensive study on how to correctly calculate the stress present in a nanofiber membrane [24].

The stiffness of the membranes is derived from the slope of the linear part present in the 0–2% range of the tensile test.

### 2.5. Digital Image Correlation (DIC)

Specimens to be analyzed by DIC were recorded using a digital microscope (Dino lite LWD AM4515ZTL). DIC was used to determine the strain field present throughout the whole sample, and these images were analyzed via the VIC-2D software. Therefore, a speckle pattern was applied on the membrane surface, using black spray paint. This did not seem to lead to a different behavior. Although the digital microscope images were corrected for lens distortions, the use of only one camera limits the accuracy of the DIC analysis if out-of-plane movements occur. Therefore, its use is only qualitative, and the samples were preloaded to ensure they laid in the imaging plane.

## 3. Results and Discussion

### 3.1. Deformation and Failure Behavior of Pristine Nanofiber Membranes

**Figure 2:**
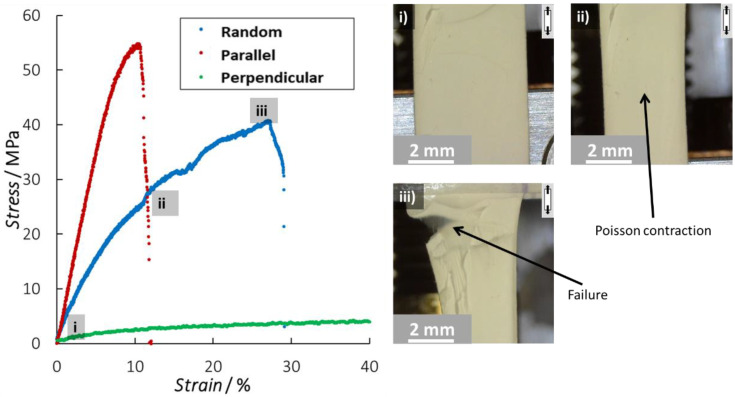
Representative tensile tests on pristine PA6 nanofiber membranes, combined with images of the progressive failure stages of a random nanofiber membrane. Figures (**i**–**iii**) show the deformation of the membrane at different stages of the stress–strain curve. Similar deformation behavior is observed for the samples with parallel and perpendicular aligned nanofibers (see Supporting Information Figure S1). No fracture was obtained for the perpendicular samples due to the travel limits of the used tensile stage.

To gain detailed insights into the deformation behavior of nanofiber non-wovens, tensile tests as described in Section 2.3 were performed on as-spun PA6 membranes. In the first phase, pristine samples with different nanofiber orientations, i.e., random, parallel, and perpendicular oriented compared to the tensile direction, were studied. For each of the orientations, one representative stress–strain curve is shown in Figure 2. The stress–strain diagrams for the three orientations differ considerably, highlighting the effect of the underlying fibrous microstructure. The higher the fraction of nanofibers that are oriented parallel to the loading direction, the higher the stiffness (Table 1, *E*). Almost twice as high stiffness values are observed for the parallel aligned membranes compared to the random oriented ones (753 ± 11 MPa vs. 370 ± 34 MPa). Furthermore, additional higher ultimate strength values (55 ± 1 vs. 39 ± 6 MPa) are obtained while the strain is reduced (12.0 ± 0.1% vs. 30.0 ± 2.8%). On the contrary, the perpendicular aligned nanofiber samples have much lower modulus values (24.1 ± 3.7 MPa) since no fibers are aligned in the tensile direction. The low stiffness is thus predominantly an effect of the cohesion of the nanofibers inside the membrane and a few fibers that are not aligned fully transversally. At the same time, much higher strain levels are obtained (>40%; note that no fracture was observed for these samples due to travel limitations of the used tensile stage).

Independent of the underlying nanofiber orientation, all the specimens show a coherent deformation behavior at the macroscale which is somewhat comparable, at least visually, to the typical characteristics of a continuous film-like material. Indeed, the specimens show a transverse contraction that is similar to the Poisson contraction observed, for example, in a continuous material. Moreover, the stress–strain curves show an almost linear elastic behavior at low tensile strains. The higher the amount of fibers present in the tensile direction, the closer this behavior is to perfect linear elastic behavior. In comparison to many other fibrous materials, there is no low stiffness or inelastic regime visible at low strains coming from the initial realignment of the fibers according to the loading direction [24,41,42,43]. Even for the random and perpendicular oriented specimens, where such realignment is expected to happen directly after mechanical loading do not show this behavior. Additionally at higher elongations, the macroscopic deformation of the specimens remains similar to that of a continuous material as a homogeneous deformation of the membrane is observed. There is thus no direct macroscopic observation that the specimens have a fibrous microstructure up to the point of failure. However, as soon as the nanofiber membrane tears, the underlying fibrous nature is clearly observed by a jagged and frizzled fracture surface similar to paper. The random and parallel oriented specimens show a well-defined ultimate tensile strength, followed by rapid fracture of the specimens (Figure 2i–iii). The rupture is well-defined, and only small fractions of fiber unraveling are noticed around the fracture zone. The perpendicular oriented specimens can accommodate very high strains and could not be tested until failure due to equipment constraints.

The macroscopic observations in Figure 2 suggest a relatively high degree of “binding” present in the nanofiber non-woven, even though no specific binding step is performed during the electrospinning production. This is different from traditional non-woven manufacturing where a binding step (calendaring, chemical binder, needling, etc.) is required for the structural integrity of the non-woven. Electrospun nanofiber membranes have a large number of contacts between the individual nanofibers due to the length of the individual fibers, resulting in a large amount of fiber-to-fiber friction interactions. In addition, the small diameter of the nanofibers compared to regular textile fibers makes the amount of contact points several orders of magnitude higher for similar grammages.

**Figure 3:**
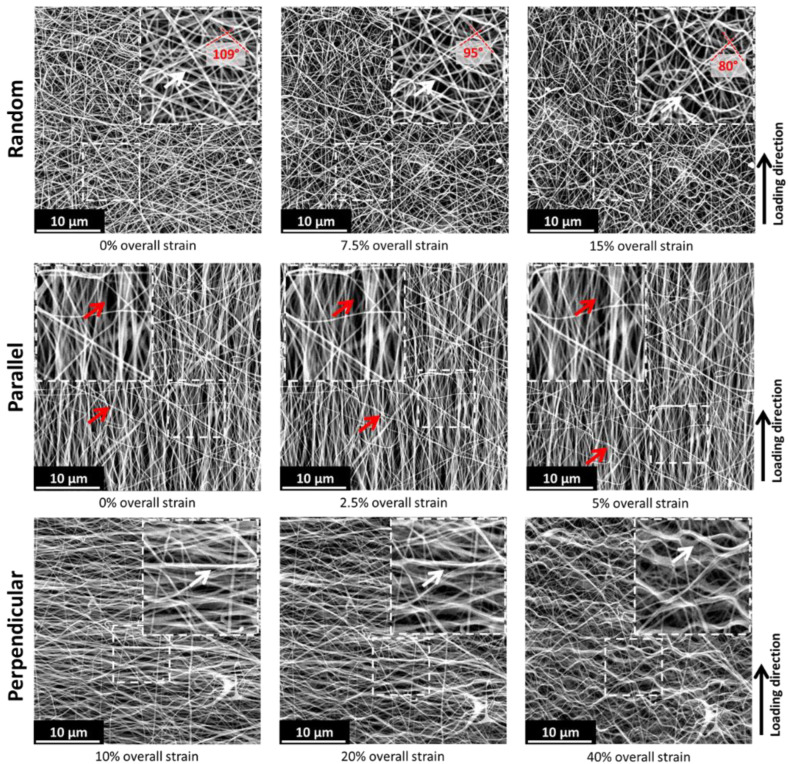
SEM images of nanofiber membranes with different fiber orientations during progressive deformation steps. Arrows indicate buckling (white) and reorientation (red) of the individual fibers.

**Figure 4:**
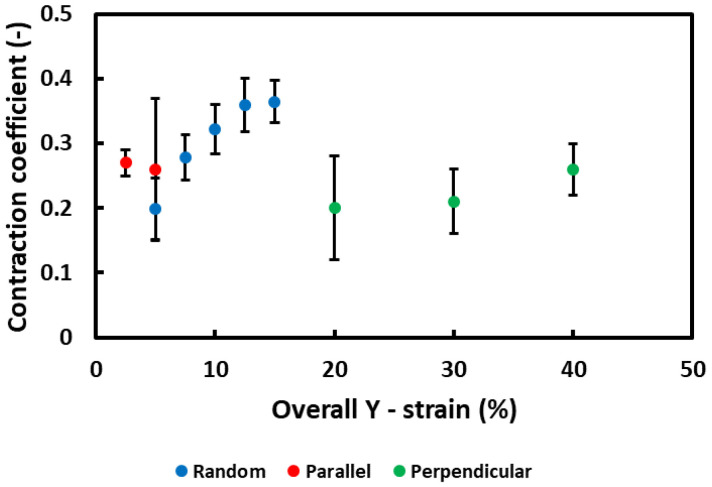
Contraction coefficient of the three different nanofiber membranes as a function of the strain. The contraction coefficient was measured based on the change in length between features in the membrane (multiple measurements on a single specimen).

SEM analysis of membranes during the testing shows the microstructural fiber displacement that results in the macroscopically observed deformation (Figure 3). Looking at the random oriented membranes, it is expected that parts of the fibers will realign themselves to the tensile direction (as is common in textiles). This is reflected in the decrease in the angle of crossing fibers (Figure 3: decrease in angle from 109° to 80°) for increasing loads. This shows the reorientation of a part of the nanofibers towards the tensile direction. At the same time, the perpendicular aligned fibers clearly show signs of buckling (waviness). The nanofibers cannot withstand the compressive loads resulting from the Poisson contraction and buckle out to accommodate these loads (Figure 3: white arrows). In the initial stage, almost no buckling is observed, and the phenomenon becomes more severe at higher strains. This progressive fiber buckling is reflected in the increasing contraction coefficient of the membrane. After the majority of perpendicular fibers are buckled out, the contraction coefficient levels off to a more or less constant value. Based on the SEM images (taken in the center region of the sample) in Figure 3, the evolution of the local contraction coefficient was calculated from the microscopically visible contraction in order to show the changing level of perpendicular contraction and elongation throughout deformation. For the purpose of clearly observing the deformation behavior in the aligned membranes, a thinner membrane (7.5 g/m^2^, 200 ± 50nm) was used to exaggerate the present effects (Figure 4: 2.5%, 0%, and 10% strains were used as reference strains for random, parallel, and perpendicular, respectively. Several measurements were performed on a single test specimen). An increase in the contraction coefficient upon increasing the y-strain is observed. This is in accordance with the increased amount of buckling at higher strains. This changing contraction coefficient shows again the unique behavior of these nanofiber membranes.

For the parallel aligned membrane, the reorientation of nanofibers is more limited (Figure 3: parallel). This can be attributed to the fact that most of these fibers are already oriented in the tensile direction. Additionally, no buckling is observed; however, some lateral contractions seem to be present. This is reflected in the decrease in the distance between the aligned fibers (red arrows). The contraction coefficient (Figure 4) calculated from the SEM images as –*ε_x_*/*ε_y_* is higher compared to the one from the random membrane. This can be related to the fact that elongation in the tensile direction is more limited since almost all fibers are already oriented in the tensile direction. An increase in standard deviation is visible at a higher strain. This is due to the small and less homogeneous perpendicular contraction.

An opposite behavior is observed for the perpendicular aligned membranes. Here almost no reorientation of the fibers is observed, but severe buckling is present (Figure 3: perpendicular, white arrows). This severe buckling seems to ‘open up’ the structure and is likely the reason for the very high strains that are observed (>40%). The fact that even for the perpendicular oriented samples the membranes do not immediately unravel shows a high degree of interconnection and entanglement between the individual nanofibers. The high strains accompanied by the buckling of the fibers is reflected in the lower contraction coefficient for the perpendicular aligned membrane (Figure 4). A slight increase in contraction coefficient is observed with the increased strain, which reflects the buckling of the fibers. At first, the fiber buckling results primarily in elongation, whereas in a later stage a large contraction perpendicular to the tensile direction is also present. In addition, a decrease in standard deviation is observed at higher strains. The deformation in the first stages is not completely homogeneous, as the buckling initiates locally and gradually occurs throughout the membrane resulting in a more uniform contraction deformation. Clearly, the orientation of the nanofibers compared to the loading direction plays an essential role in the deformation of the membrane.

### 3.2. Influence of Damage on the Deformation Behavior

**Figure 5:**
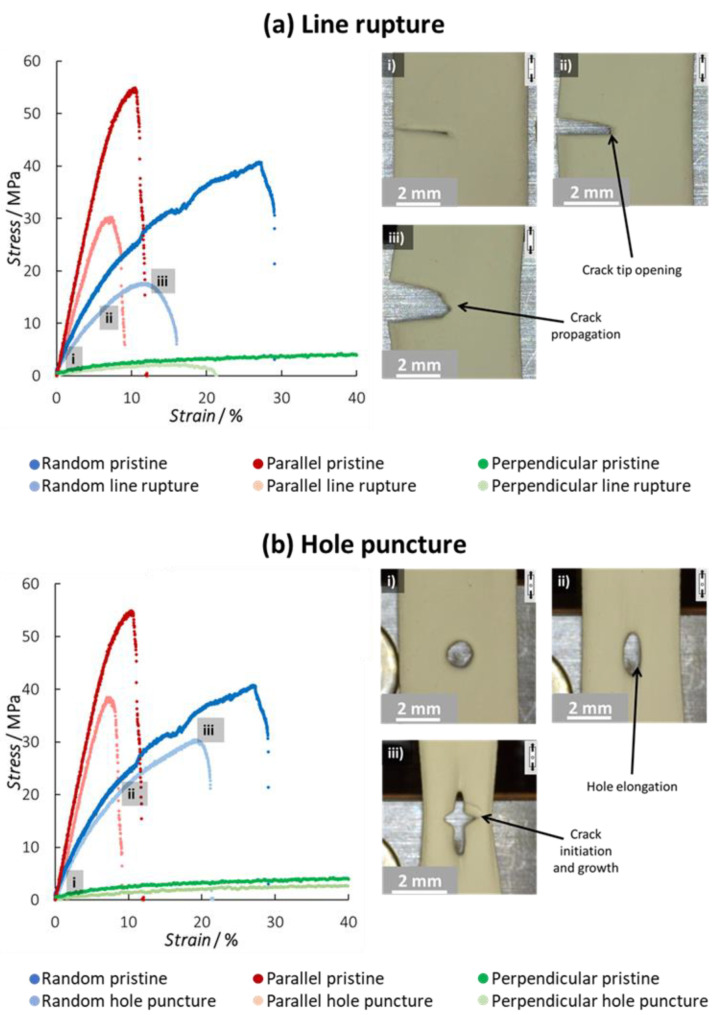
(**a**) Overview of representative tensile tests on nanofiber membranes with a line rupture, combined with images of the progressive failure stages of a random nanofiber membrane. (**b**) Overview of representative tensile tests on nanofiber membranes with a hole puncture, combined with images of the progressive failure stages of a random nanofiber membrane. Figures (**i**–**iii**) show the deformation of the membrane at different stages of the stress–strain curve. Similar deformation behavior is observed for the samples with parallel and perpendicular aligned nanofibers (see Figures S2 and S3 in the Supporting Information).

To understand the effect of membrane damage on the micro- and macroscale deformation behavior of the nanofiber membranes, the same tensile tests were performed on pre-damaged samples that included a cut at one side or a hole in the center (see Section 2.2). Both the cut and the hole lower the effective cross-sectional area taking up the load, resulting in a decreased nominal tensile stress compared to the undamaged specimens (Table 2). Furthermore, possible stress concentrations at the tip of the damaged zone and less restriction of fiber movement may also influence the mechanical behavior of the membranes. These pre-imposed damages also affect the failure mechanism of the membrane as they cause a stress concentration. The line rupture seems to have a detrimental effect on the stiffness of the nanofiber membranes for all fiber orientations (Figure 5a: dark vs. light curves). Whereas the hole rupture seems to have a less negative effect on the stiffness of the membranes (Figure 5b). A good cohesion of the nanofibers in the membranes (due to many contact points) possibly redistributes the stress around the hole during the initial stage of the tensile tests. However, the drop in ultimate tensile stress and strain shows that both the line rupture and hole puncture influence the mechanical properties of the membrane.

For the specimens containing a cut, independent of the fiber orientation in the specimens, the crack tip opens in the loading direction and becomes blunter without the crack propagating into the membrane in the first stage of the test (Figure 5a). This again shows that the nanofiber membranes have inherently good structural integrity. At a critical load level, the crack starts to propagate through the sample. At this point, a gradual deflection from the linear slope on the stress–strain diagram is observed. The crack then progresses relatively stable throughout the remaining ligament of the specimen, especially in comparison to the undamaged specimens where a more brittle fracture is observed (e.g., steeper drop in Figure 5a). This macroscopic behavior is similar to that of thermoplastic polymer films during Single Edge Notch Testing [44]. Similar to the pristine specimens, the underlying nanofiber orientation does have a strong effect on the stress levels reached in the specimens, where a higher fiber orientation along the tensile direction results in a stiffer response.

For the punctured specimens, the behavior is very similar. Independently of the fiber orientation, the hole elongates in the tensile direction. At a critical load level, cracks appear at the side of the hole where there is a stress concentration. This goes hand in hand with a deflection from the linearity of the slope on the stress–strain diagram. The crack progresses relatively stable throughout the remaining ligament for a certain crack length, after which the specimen fails abruptly. Again, the fiber orientation affects the stiffness of the specimens considerably, while the overall macroscopic deformation remains more or less similar.

**Figure 6:**
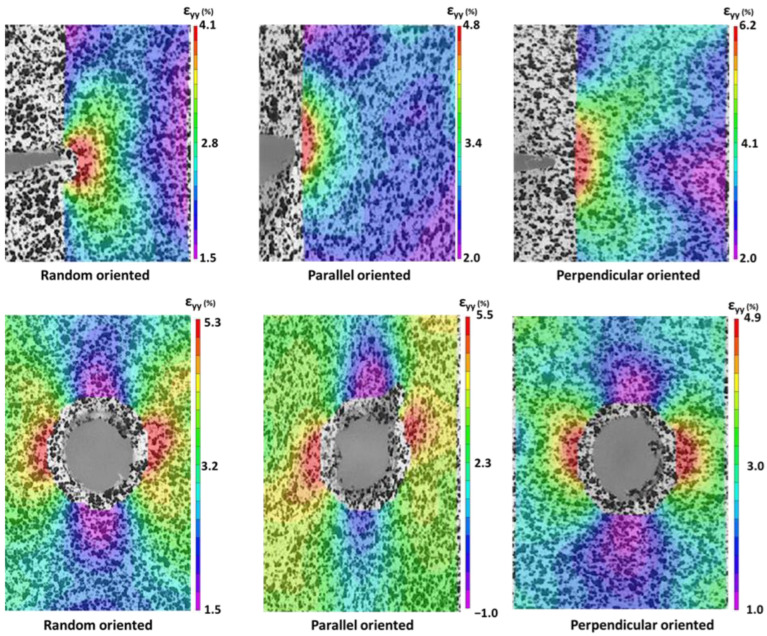
DIC inspection of the strain (y-direction) present around a notch for a random, parallel, and perpendicular oriented membrane.

More detailed insights into the local deformation behavior around the pre-imposed damage are obtained using DIC (Figure 6). Overall, the nanofiber membranes show similar strain fields, especially for the random oriented membranes. For the parallel and perpendicular oriented specimens, the strain concentrations can be seen to be more elongated along the fiber direction. For example, in the cut specimen with parallel oriented nanofibers, the lobes at the crack tip are not so apparent, and the strain concentration seems to be almost completely smeared out axially, resulting in a more circular pattern around the crack tip.

The DIC results show that there is good strain transfer (and thus also stress transfer) in the nanofiber membranes, even when there is a sharp crack/cut present. Even for parallel oriented membranes, where nanofibers are aligned next to each other in the loading direction, the stress is distributed laterally away from the crack tip, albeit to a much smaller extent than the random and perpendicular aligned membranes. This indicates that the strain (and thus stress) is mainly diverted in the direction of the nanofibers. The strain fields are thus similar to those of a (linear elastic) solid material, although the influence of the underlying nanofiber orientation is clearly visible.

**Figure 7:**
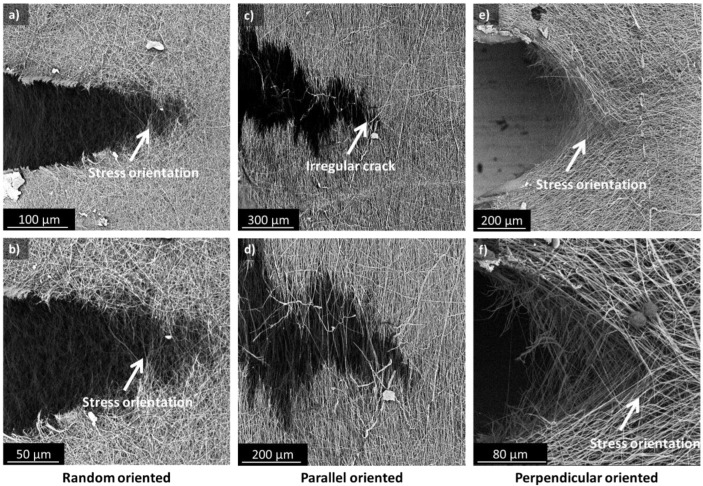
In situ SEM observations at the crack tip for random (**a**,**b**), parallel (**c**,**d**), and perpendicular oriented nanofiber membranes (**e**,**f**).

A closer look at the crack tip of the pre-cut specimens using in situ SEM learns that the fibers reorient at the microscale and are pulled out in the loading direction locally (Figure 7). This reorientation and pull-out only take place at the crack tip itself; further away, the membrane behaves like an undamaged sample. Upon crack opening, nanofibers span both sides of the crack tip and realign until they are almost parallel with the tensile direction. This mechanism restricts crack growth as the fibers need to be broken before the crack can propagate, which gives the membrane a high toughness. As a result, the random and perpendicular oriented membranes have a smoother crack path as there is a high amount of fiber realignment taking place. Whereas, for the parallel aligned membranes, the crack path is less regular due to the already oriented nanofibers that will break at their weakest spot (Figure 7c–d). Note that even the perpendicular oriented membranes can withstand some loading before the crack propagates throughout the sample. Since all fibers are aligned parallel to the cut, one could expect that these samples immediately break when loading is applied. However, the membrane withstands small loadings due to the cohesion, entanglement, and imperfect alignment of all the nanofibers. This gives the nanofibers the possibility to reorient in the stress direction nearby the crack tip (Figure 7e–f). This prevents the direct propagation of the crack, and thus initially keeps the membrane together.

## 4. Conclusions

Mechanical tests on solvent electrospun polyamide 6 nanofiber membranes showed that these membranes behave as a coherent structure even without the presence of a binder. This is mainly due to a large number of intimate contact points between the individual nanofibers in the membrane. During the first stages of deformation, the membranes behave comparably to regular film material; this is no longer the case upon larger deformation. At higher strain levels, the internal rearrangement and buckling of nanofibers lead to specific macroscopic deformation behavior. The mechanical properties could be tuned by aligning the nanofibers parallel (high stiffness and strength; low strain) or perpendicular (low stiffness and strength; high strain) to the load direction. Overall, all membranes, both un- and pre-damaged showed a coherent behavior up to the point of fracture. This clearly shows the potential to use polymeric nanofiber membranes as tunable stand-alone material. Indeed, even when damaged, these membranes show their integrity and resistance to failure. The nanofibers in the proximity of a stress concentration (line rupture or hole) distribute the stress throughout the sample and even align themselves in the stress direction, increasing their resistance against further deformation. Similar behavior was observed around the hole of the punctured nanofiber membranes. These polymeric nanofiber membranes can thus cope with damage up to a certain point. This again shows their potential to be used as stand-alone materials.

## Figures and Tables

**Table 1:** Overview of the mechanical data of all tested membranes. The strength is determined based on the nominal cross-section of the specimens.

Nanofiber Membrane		E	$\sigma_{UTS}$	$\varepsilon_{b}$
Orientation	Damage	(MPa)	(MPa)	(%)
Random	None	370 ± 34.1	38.5 ± 6.0	30.0 ± 2.8
Parallel	None	753 ± 11.3	55.4 ± 0.8	12.0 ± 0.1
Perpendicular	None	24.1 ± 3.7	4.4 ± 0.6 *	/ *

*: maximum clamp displacement was reached before fracture.

**Table 2:** Ultimate tensile strength for pristine and damaged nanofiber membranes.

Nanofiber Membrane		E	$\sigma_{UTS}$	$\varepsilon_{b}$
Orientation	Damage	(MPa)	(MPa)	(%)
Random	Line rupture	238 ± 15.0	16.5 ± 1.3	15.3 ± 1.1
	Hole puncture	379 ± 18.0	31.0 ± 0.6	22.1 ± 1.1
Parallel	Line rupture	492 ± 50.0	29.2 ± 0.9	9.6 ± 0.5
	Hole puncture	703 ± 76.0	37.1 ± 1.1	8.2 ± 0.6
Perpendicular	Line rupture	18.3 ± 3.8	2.1 ± 0.1	25.0 ± 2.6
	Hole puncture	17.6 ± 3.1	4.1 ± 0.3 *	/ *

*: maximum clamp displacement was reached before fracture.

## Data Availability

The data presented in this study are available on request from the corresponding author.

## Supplementary Materials

The following supporting information can be downloaded at: https://www.mdpi.com/article/10.3390/polym15071630/s1, Figure S1: Overview of all stress–strain curves grouped per orientation and damage type. Figure S2: Subsequent deformation images of random, parallel, and perpendicular oriented membranes. Similar behavior is observed for the three different kinds of membranes. Figure S3: Subsequent deformation images of random, parallel, and perpendicular oriented membranes pre-damaged with a line rupture. Similar behavior macroscopic behavior of crack opening and propagation is observed up to the point of final fracture. Figure S4: Subsequent deformation images of random, parallel, and perpendicular oriented membranes pre-damaged with a puncture. Similar behavior macroscopic behavior of hole elongation, crack initiation, and propagation is observed. Figure S5: Subsequent SEM deformation images of a pristine random oriented nanofiber membrane. Figure S6: Subsequent images of a pristine parallel oriented nanofiber membrane.
