# Supplementary material for: Microscale and Macroscale Deformation Behavior of Electrospun Polymeric Nanofiber Membranes Using In Situ SEM during Mechanical Testing
Source: Polymers (Basel). 2023 Mar 24;15(7):1630. doi: 10.3390/polym15071630 (PMC10096881; doi:10.3390/polym15071630)
Supplement: Supplementary file 1 [file polymers-15-01630-s001.zip › polymers-2218459-supplementary.pdf]

Microscale and macroscale deformation behavior of electrospun polymeric nanofiber membranes using in-situ SEM during mechanical testing

Olivier Verschatse, Eva Loccufier, Bianca Swanckaert, Karen De Clerck and Lode Daelemans*

Department of Materials, Textiles and Chemical Engineering (MaTCh), Faculty of Engineering and Architecture, Ghent University, Technologiepark 70A, 9052 Ghent, Belgium

* *Correspondence: Corresponding author: Lode.Daelemans@UGent.be*

Supporting information

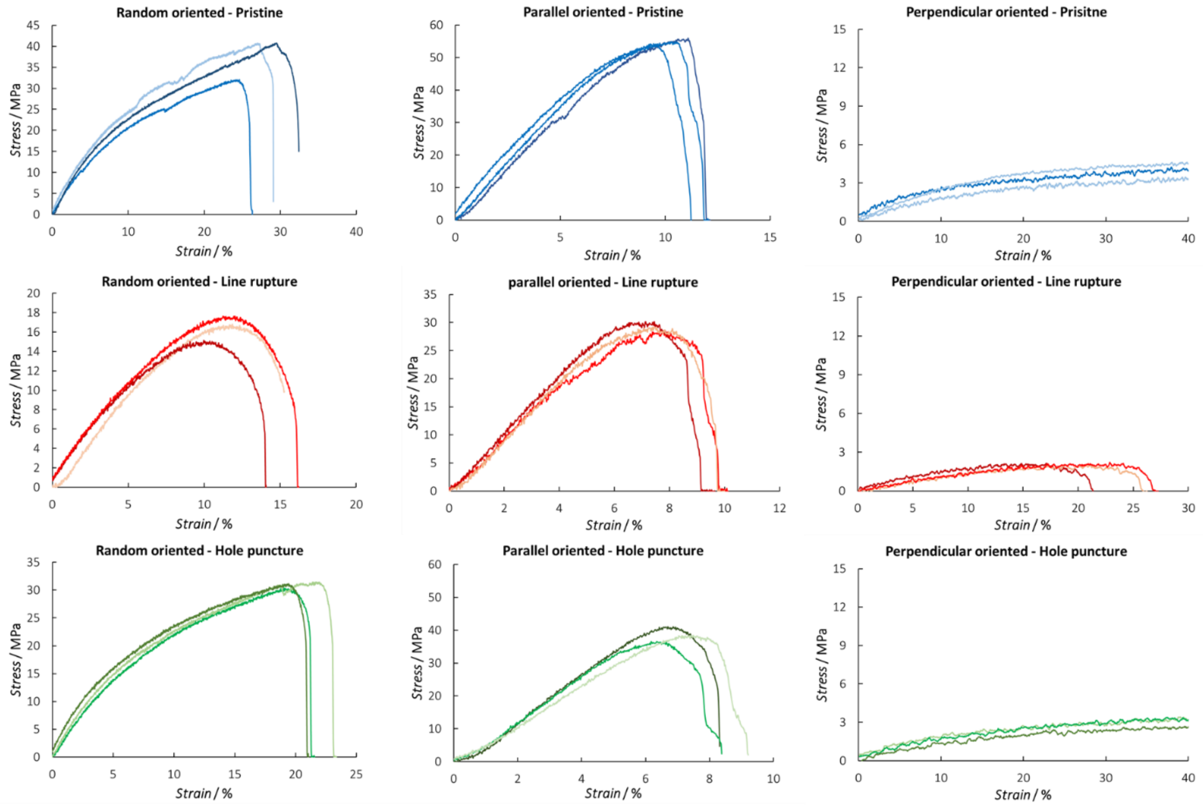

Figure S1: Overview of all stress strain curves grouped per orientation and damage type.

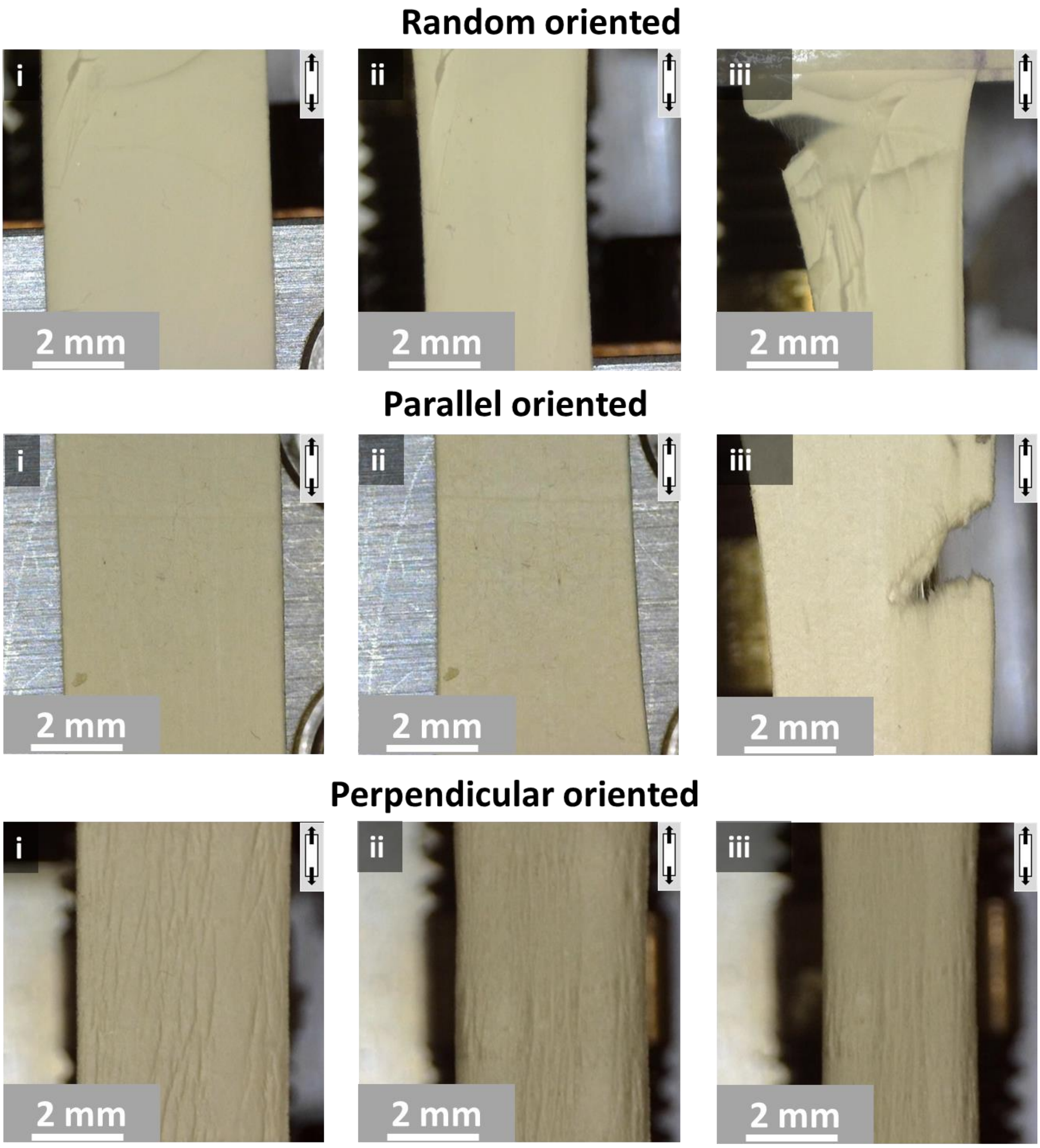

Figure S2: Subsequent deformation images of random, parallel, and perpendicular oriented membranes. Similar behavior is observed for the three different kinds of membranes.

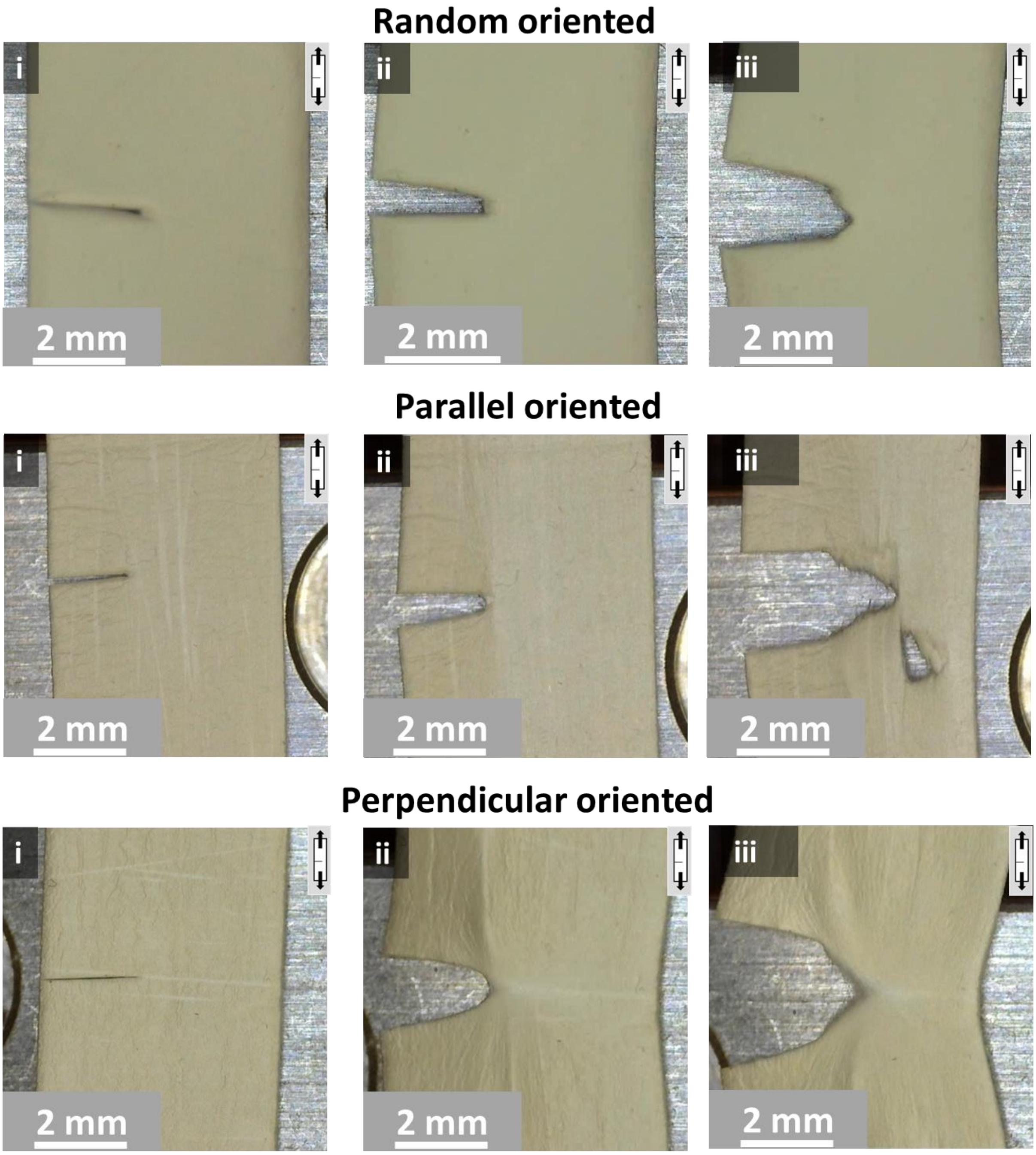

Figure S3: Subsequent deformation images of random, parallel, and perpendicular oriented membranes pre-damaged with a line rupture. Similar behavior macroscopic behavior of crack opening and propagation is observed up to the point of final fracture.

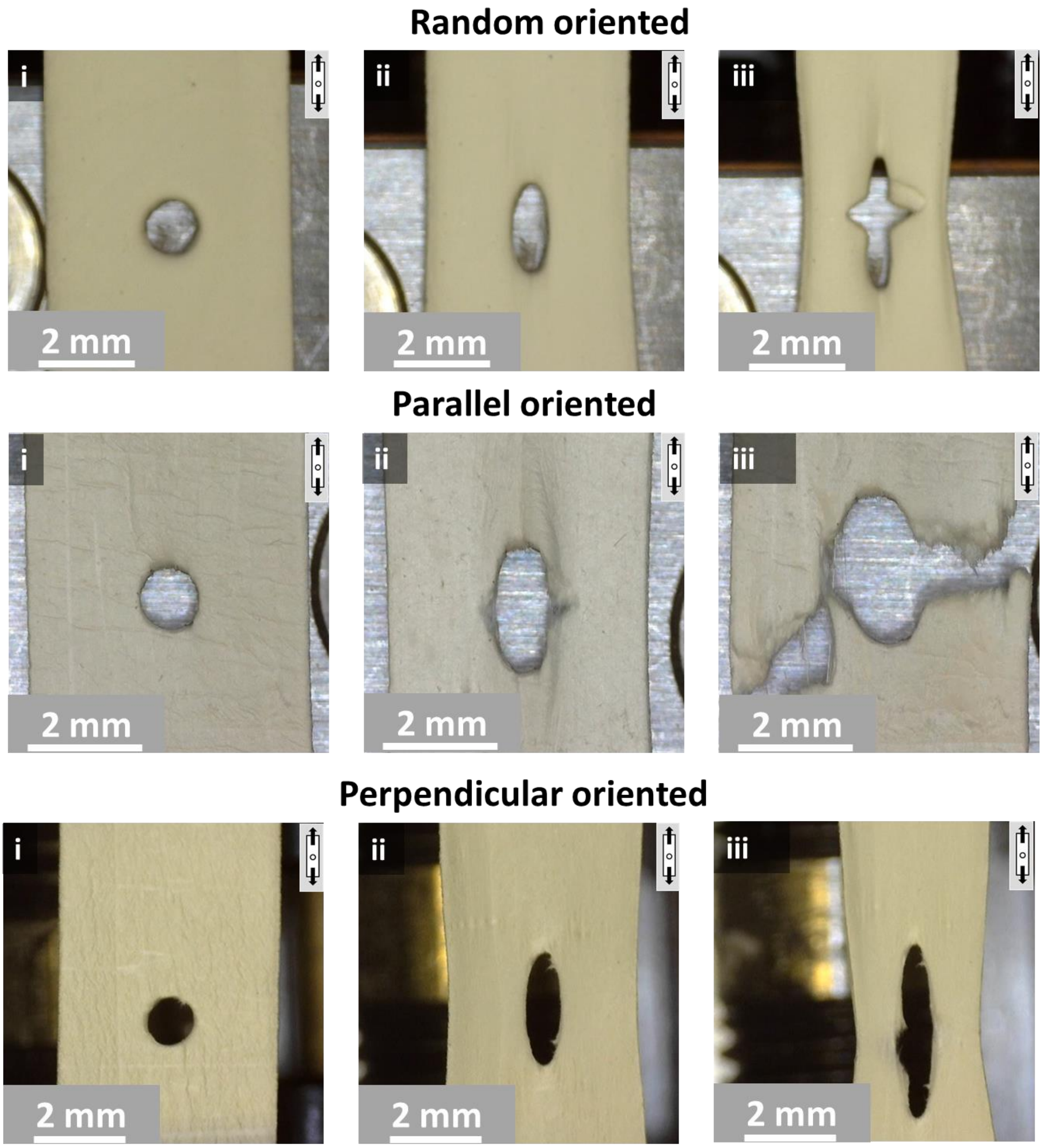

Figure S4: Subsequent deformation images of random, parallel, and perpendicular oriented membranes pre-damaged with a puncture. Similar behavior macroscopic behavior of hole elongation, crack initiation, and propagation is observed.

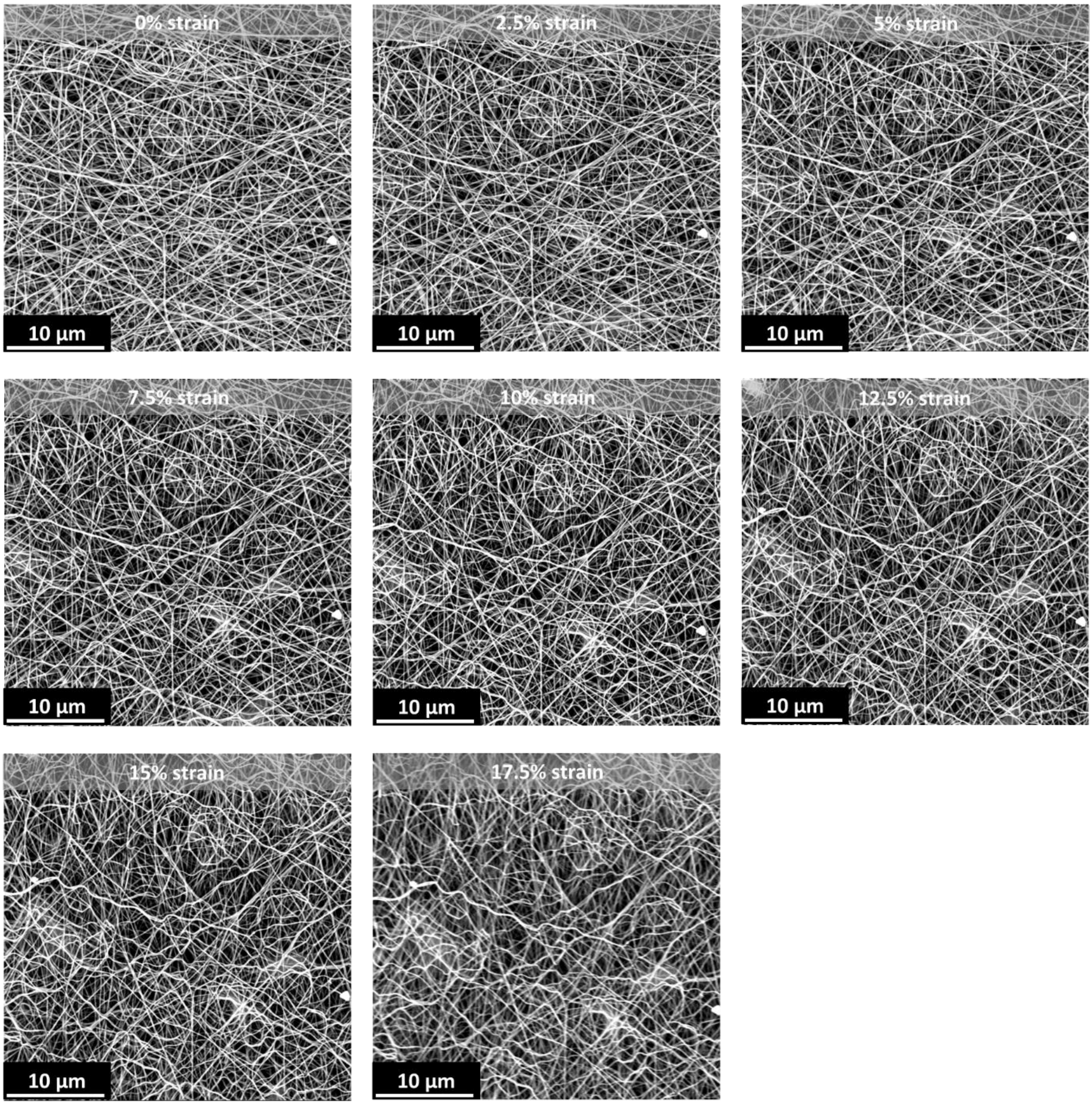

Figure S5: Subsequent SEM deformation images of a pristine random oriented nanofiber membrane.

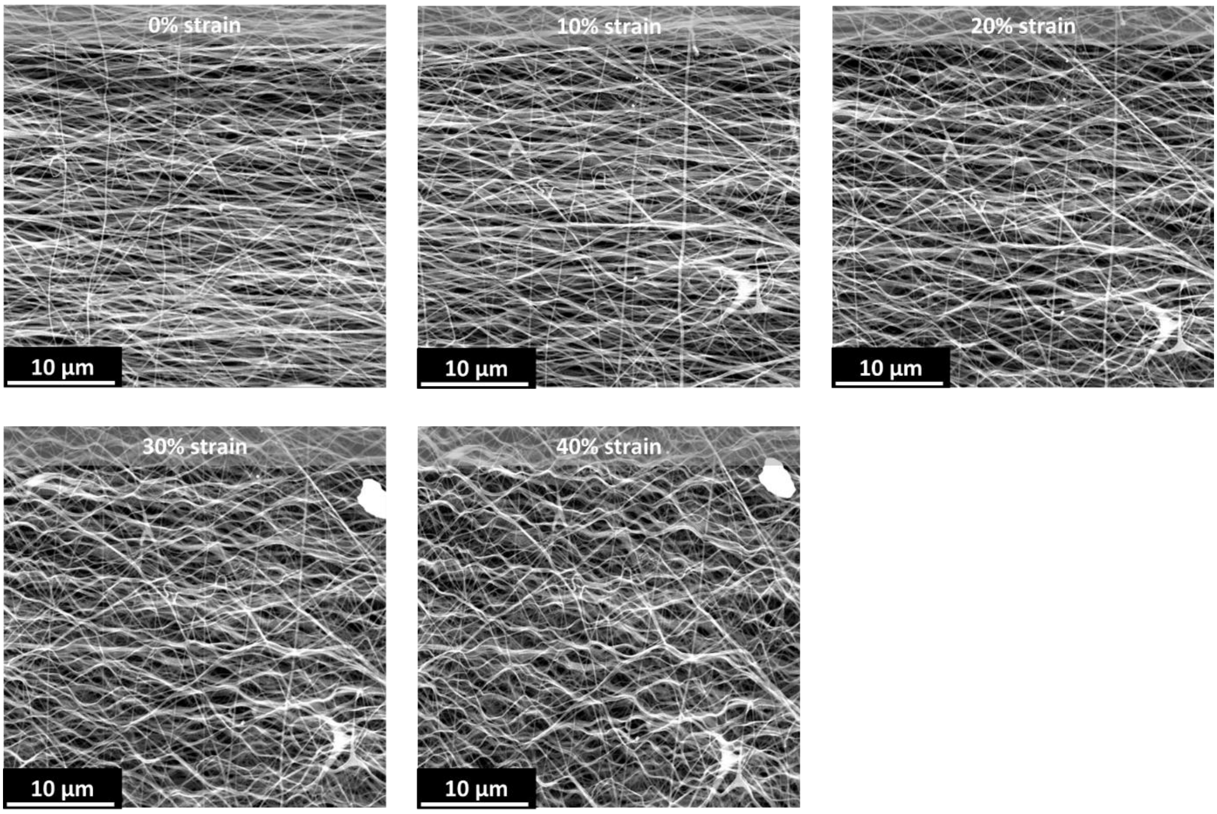

Figure S6: Subsequent images of a pristine parallel oriented nanofiber membrane.
